# Breast Cancer-Related Chemical Exposures in Firefighters

**DOI:** 10.3390/toxics12100707

**Published:** 2024-09-28

**Authors:** Bethsaida Cardona, Kathryn M. Rodgers, Jessica Trowbridge, Heather Buren, Ruthann A. Rudel

**Affiliations:** 1 Silent Spring Institute, Newton, MA 02460, USA; 2 Department of Environmental Health, Boston University School of Public Health, Boston, MA 02118, USA; 3 Department of Obstetrics Gynecology and Reproductive Sciences, University of California San Francisco, San Francisco, CA 94143, USA; 4 United Fire Service Women, San Francisco, CA 94140, USA

**Keywords:** benzene, female firefighter, flame retardant, per- and polyfluoroalkyl substance, polycyclic aromatic hydrocarbon, styrene, volatile aromatics, organohalogens

## Abstract

To fill a research gap on firefighter exposures and breast cancer risk, and guide exposure reduction, we aimed to identify firefighter occupational exposures linked to breast cancer. We conducted a systematic search and review to identify firefighter chemical exposures and then identified the subset that was associated with breast cancer. To do this, we compared the firefighter exposures with chemicals that have been shown to increase breast cancer risk in epidemiological studies or increase mammary gland tumors in experimental toxicology studies. For each exposure, we assigned a strength of evidence for the association with firefighter occupation and for the association with breast cancer risk. We identified twelve chemicals or chemical groups that were both linked to breast cancer and were firefighter occupational exposures, including polycyclic aromatic hydrocarbons, volatile aromatics, per- and polyfluoroalkyl substances, persistent organohalogens, and halogenated organophosphate flame retardants. Many of these were found at elevated levels in firefighting environments and were statistically significantly higher in firefighters after firefighting or when compared to the general population. Common exposure sources included combustion byproducts, diesel fuel and exhaust, firefighting foams, and flame retardants. Our findings highlight breast-cancer-related chemical exposures in the firefighting profession to guide equitable worker’s compensation policies and exposure reduction.

## 1. Introduction

Compared to the general population, firefighters have an increased cancer incidence [1,2,3,4,5,6,7,8] and mortality [1,9,10,11]. The increased risk has been associated with cumulative firefighting chemical exposures [12] and increased length of employment as a firefighter [6]. The International Agency for Research on Cancer (IARC) has classified occupational exposure as a firefighter as carcinogenic to humans (Group 1) [13] due to sufficient evidence of mesothelioma and bladder cancer. However, there is limited information on occupational cancer risks to firefighters that primarily affect females—such as breast cancer—because firefighting has historically been a profession held predominantly by males. Indeed, some studies on firefighter cancers have excluded females because of small numbers [6,14] or advised caution and suggested that additional analyses are needed to confirm study results [1,3,9,11,15]. Addressing the data gap on health risks to female firefighters has become more urgent as the number of female firefighters increases [16,17].

The few studies investigating breast cancer risk among firefighters suggest an increased risk. For example, a US study using data from 1950 to 2009 found that female firefighters had a 39% increased risk of dying from breast cancer compared to women in the general US population and a 26% increased risk of being diagnosed with breast cancer compared to women in the US population [1]. In a follow-up study adding 16 years of observation, mortality from breast cancer among female firefighters remained elevated when compared to the general population, showing a 41% increased risk [10]. However, neither of these findings reached statistical significance, which the authors suggested was because the cohort included less than 4% women [1,10]. Despite the rarity of breast cancer in males, male firefighters may also have higher levels of breast cancer. A study of Florida firefighters from 1972 to 1999 found that male firefighters had 7.4 times the risk of dying from breast cancer compared to males in Florida’s general population [9]. A study of male Australian firefighters reported statistically significantly increased breast cancer incidence among full-time firefighters on the job for 20 years or more [4]. While both findings are based on a small number of breast cancer cases among men, these results are striking because of the rarity of male breast cancer [18,19]. It is important to note that bias associated with the healthy worker effect means that these studies could underestimate the true excess risk [20].

Overall, firefighters are exposed to vast numbers of toxic chemicals through their work [21,22], and many of these exposures are anticipated to contribute to breast cancer risk. For example, chemicals that firefighters are exposed to, such as polycyclic aromatic hydrocarbons (PAHs), fossil fuel components, and flame retardants, have been linked to breast cancer risk in epidemiological studies or shown to increase mammary tumors in experimental toxicology studies [23,24,25,26]. Over the past 50 years, furnishings have been increasingly made of oil-based plastics and other synthetic materials (which burn faster, hotter, and produce more toxic smoke [27]), include more flame retardants, and produce more toxic byproducts when burned [21].

Because breast cancer is so prevalent among females [28], identifying exposures that increase risk is a public health priority. To better understand firefighter exposures to potential breast carcinogens, we conducted a systematic search and review [29] to identify firefighter chemical exposures and then cross-referenced the resulting list with evidence about whether those chemicals influence breast cancer. With an estimated 89,600 (9%) female firefighters in the United States in 2020 [30], it is urgent to identify chemical exposures that may increase breast cancer risk to guide exposure reduction. Firefighters are also eligible for worker’s compensation benefits when they contract work-related illnesses, so identifying exposures that increase breast cancer risk can guide fair compensation policies.

## 2. Materials and Methods

In the present study, we systematically searched two different topic areas and compiled evidence at their intersection. Specifically, we used existing reviews to identify chemical exposures associated with breast cancer in humans or mammary tumors in experimental studies, and we also systematically searched to identify chemical exposures associated with the firefighting occupation. We then brought these together, identifying firefighter exposures that were also breast-cancer-relevant chemicals. We assigned a strength of evidence classification to each dimension—in other words, we classified evidence of elevated firefighter exposures as strong or inadequate, and likelihood of breast cancer following adult chemical exposure as probable, moderate, and limited. Based on the review categories described by Grant and Booth (2009), we describe our approach to identifying firefighter exposures to breast cancer related chemicals as a systematic search and review [29]. We used the Population, Exposure, Comparison, and Outcome (PECO) framework to generate the following research question and guide our review: Do firefighters (P) have exposures to environmental chemicals (E) that are noted to be either elevated or measured/detected in firefighters or at the fire scene (C) and that are identified in other studies as being breast-cancer-relevant (O).

### 2.1. Breast-Cancer-Relevant Exposures

To identify firefighter chemical exposures that were breast-cancer-relevant, we compiled a list of 301 breast-cancer-relevant chemical exposures (Supplementary Table S1). We derived this list from two sources. The first was a study identifying 278 chemicals that caused mammary gland tumors in rodents [26] based on information compiled from sources such as IARC and the US National Toxicology Program (NTP). The second source was a review of epidemiological evidence of breast cancer risk that included 158 peer-reviewed articles published between 2006 and 2016 in PubMed [23]. From this review, we identified 30 chemicals or chemical groups that were linked to breast cancer in epidemiological studies, with 9 of these chemicals also included in the list of chemicals that induced mammary gland tumors in rodents. To capture epidemiological evidence since the Rodgers et al. 2018 review, we conducted a search for studies published from June 2016 to June 2022 that included chemical exposures that we defined (below) as having adequate evidence of exposure through the firefighting occupation. We reviewed these newer studies to assess whether the evidence of breast cancer associations had changed since the 2018 review. The search terms, number of studies identified, and articles reviewed are provided in Supplemental Tables S2 and S3.

For those breast-cancer-relevant chemicals with elevated exposure in firefighters (defined below), we classified the likelihood of breast cancer following adult exposure (most relevant to firefighters) as probable, moderate, or limited based on the following criteria: probable = either strong evidence of breast cancer in humans or suggestive evidence in humans and consistent evidence of mammary tumors in rodents; moderate = either consistent evidence of mammary tumors in rodents or suggestive evidence of breast cancer in humans but limited evidence of mammary tumors in rodents; limited = inconsistent evidence of breast cancer and inconsistent evidence of mammary tumors in rodents. Because of the chemical diversity of flame-retardant chemicals, we separated them into three classes: polybrominated diphenyl ethers (PBDEs), other brominated flame retardants (BFRs), and organophosphate flame retardants (OPFRs).

### 2.2. Firefighter Exposures

**Figure 1:**
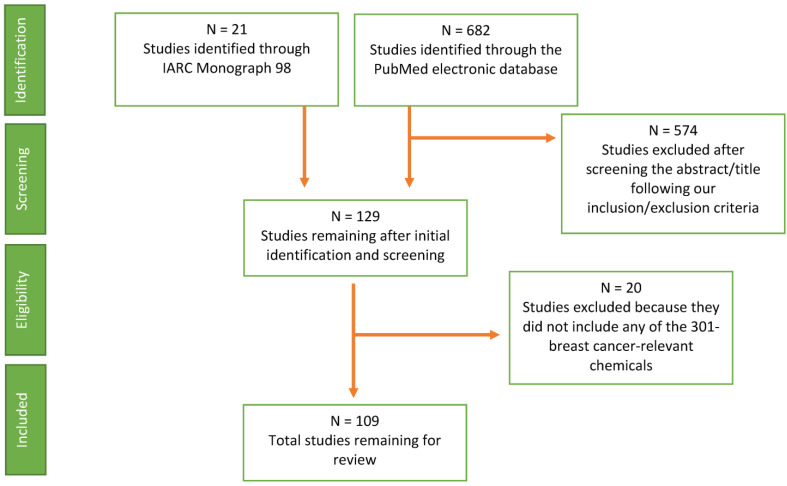
Flow chart of article selection for identifying firefighter exposures.

We identified articles documenting firefighter chemical exposures by conducting a literature search using the PubMed database (Figure 1). We searched for articles with the keywords “firefighter(s)” and “exposures(s)” in the title or abstract that were published between January 2005 and May 2022 (when we conducted the search) (Supplemental Table S4). The article PubMed Identifiers were uploaded to abstrackr [31], an open-source and web-based tool that allowed us to manually screen the article abstract for relevance while machine learning tools ran in the background to semi-automate the screening process. During this screening phase focusing on the title and abstract, we applied the following inclusion/exclusion criteria: (1) primary article and not a review or meta-analysis; (2) chemical exposures were measured (we did not include other relevant firefighter exposures such as stress or shiftwork); and (3) focus on firefighter populations in general, rather than on case studies or catastrophic events that might not be generalizable to other firefighters; for example, we excluded studies on the World Trade Center or Notre Dame Cathedral fire. We included studies of training scenarios because they were relevant to firefighter exposures. The authors BC and KR screened articles until abstrackr predicted that none of the remaining articles were relevant (N = 310), after which we downloaded the PDF files of the articles that passed our screening criteria for further review (Supplemental Table S5).

In addition to the articles identified through the PubMed database, we included articles identified in the IARC Monograph 98 [21] as reporting potential firefighter exposure to any of the breast-cancer-relevant chemicals (Figure 1, Supplementary Table S4). IARC Monograph 98 (whose working group convened in 2007) was a source for findings published prior to 2005, while our PubMed literature search only included articles after 2005.

Having identified articles that documented firefighter chemical exposures (Supplementary Table S5) we further narrowed the review to those articles that included any of the 301 breast-cancer-relevant chemicals, or their synonyms as identified through a CompTox Dashboard batch search (see Supplementary Table S1 for a full list of chemicals used and their synonyms). We used text mining tools on the full text in R version 4.1.0 to link each of the firefighter exposure articles with the breast-cancer-relevant chemical they included (Supplemental Table S6). We noted the number of articles that included each of the breast-cancer-relevant chemicals (Table 1).

For each of the breast-cancer-relevant chemicals included in an article on firefighter exposures, we noted whether the original study authors reported elevated chemical exposure levels in firefighters. We subsequently labeled the evidence of elevated exposure in firefighters as “strong”, “some”, or “inadequate”. Chemicals labeled as having strong evidence of exposure in firefighters were those noted by at least one of the original studies as being statistically significantly elevated in firefighters (comparing firefighters to non-firefighters or firefighters before and after a firefighting exposure, for example through biomonitoring, wipe sampling, or personal air sampling) or in the firefighter environment (study authors reported concentrations above an occupational guideline). Chemicals labeled as having some evidence of elevated firefighter exposure were those that were consistently detected or measured in firefighters or the firefighter environment but did not meet the criteria for elevated exposure. Finally, chemicals labeled as having inadequate evidence of elevated exposure in firefighting were those that were only identified as elevated in a single study or had inconsistent detections, meaning that they were detected in some instances, often at low levels, but not in others.

## 3. Results

### 3.1. Articles Selected

Through PubMed, we identified 682 articles that contained the keywords “firefighter(s)” and “exposure(s)” in the abstract or title, in publications between January 2005 and May 2022 (Figure 1; Supplemental Table S4). Using abstrackr, we screened titles and abstracts based on the exclusion/inclusion criteria previously described and identified 108 potentially relevant articles (Supplemental Table S5). We supplemented these articles with 21 studies reviewed in the IARC Monograph 98 (Supplemental Table S5). This yielded a total of 129 articles relevant to firefighter exposures (Figure 1). Finally, we screened these 129 articles for any mention of the 301 breast-cancer-relevant chemicals or chemical groups that induced mammary tumors in rodent studies or were linked to breast cancer in epidemiological studies, yielding 109 articles that identified firefighter exposures linked to breast cancer (Figure 1).

### 3.2. Firefighter Chemical Exposures Identified as Breast-Cancer-Relevant

There were 28 breast-cancer-relevant chemicals or chemical groups in the firefighter exposure studies. We combined OPFRs and BFRs into one category since these are both replacement flame retardants for PBDEs. Table 1 summarizes our assessment of the evidence for each of these exposures associated with firefighters and with breast cancer. Table 2 includes brief summaries of the studies that we evaluated to make these assessments. Nine of these twenty-eight chemicals, including benzene, dioxin-like compounds, PBDEs, and PAHs, were considered to have strong evidence of elevated exposure in firefighting (Table 1) because chemical levels were noted by at least one study author as exceeding an occupational guideline or were elevated in firefighters when compared to the general population or when comparing pre- and post-exposure levels (Table 2). Three chemicals had some evidence of elevated exposure in firefighting because they were detected in the firefighting environment or in firefighters but did not meet the criteria for elevated exposure. Sixteen of the twenty-eight chemicals identified in firefighter exposure studies were considered to have inadequate evidence of exposure in firefighters because they were only included in a single study (N = 9) or were inconsistently detected and had low concentrations when detected (N = 7).

Of the nine chemicals or chemical groups with strong evidence of firefighter exposure, we considered two to have a probable likelihood of increasing breast cancer risk following exposure in adults (Table 1). These chemicals, benzene and PAHs, both had suggestive evidence of elevated breast cancer risk based on epidemiological studies and strong evidence of inducing mammary gland tumors in experimental rodents following different methods of administration (Table 2). Three other chemicals with strong evidence of firefighter exposure—acetaldehyde, styrene, and replacement flame retardants—were considered to have a moderate likelihood of increasing breast cancer following adult exposures (Table 1). While both acetaldehyde and styrene increased the incidence of mammary gland tumors in rats, epidemiological evidence for increased breast cancer risk was limited for styrene and unavailable for acetaldehyde. Flame retardants as a class remain relatively understudied, although we have assigned the replacement flame retardants OPFRs and BFRs as having “presumed moderate” evidence for increasing breast cancer risk based on mammary tumors in rodent studies of structurally relevant chemicals: a brominated flame retardant (2,2-bis(bromomethyl)-1,3-propanediol) and direct and indirect metabolites of organophosphorus flame retardants (2,3-dibromo-1-propanol and 3-monochloropropane-1,2-diol, respectively) [32,33]. Finally, four of the nine chemicals were considered to have limited data of increasing breast cancer risk following adult exposure—including dioxins and dioxin-like compounds, PBDEs, PFASs, and PCBs. This is due to inconsistent results, of either the direction of association or statistical significance, from epidemiological and experimental studies.

All three of the chemicals identified to have some evidence of elevated exposure in firefighting, acrylonitrile, 1,3-butadiene, and isoprene, were considered to have a moderate likelihood of increasing breast cancer following adult exposure (Table 1). All three increased the incidence of mammary gland tumors in rodent studies. Epidemiological data were unavailable for acrylonitrile and isoprene and were limited to one study for 1,3-butadiene (note that butadiene is an air pollutant and there are quite a few studies of air pollution and breast cancer and other cancers). We did not assign a classification for the likelihood of breast cancer for the 16 breast-cancer-relevant chemicals with inadequate evidence of firefighter exposure.

Notably, IARC classified 8 of the 12 breast-cancer-relevant chemicals as potentially carcinogenic firefighter exposures (Table 2) [34]. IARC classified benzene, benzo[a]pyrene, 1,3-butadiene, PCBs, and 2,3,7,8-tetrachloro dibenzo-para-dioxin (TCDD) as carcinogenic to humans (Group 1), styrene as probably carcinogenic (Group 2A), and acetaldehyde and isoprene as possibly carcinogenic (Group 2B). IARC also lists several PAHs as possibly or probably carcinogenic, including dibenz[a,h]anthracene, benz[a]anthracene, benzo[b]fluoranthene, benzo[k]fluoranthene, and indeno-1,2,3-[cd]pyrene.

**Table 2:** List of breast-cancer-relevant chemicals associated with evidence of elevated exposure in the firefighters or the firefighter environment, along with brief summaries for the evidence of breast cancer and elevated firefighter exposure relevance based on the reviewed literature. A list of potential exposure sources and events for each chemical is included [35,36,37,38,39,40,41,42,43,44,45,46,47,48,49,50,51,52,53,54,55,56,57,58,59,60,61,62,63,64,65,66,67,68,69,70,71,72,73,74,75,76,77,78,79,80,81,82,83,84,85,86,87,88,89,90,91,92,93,94,95,96,97,98,99,100,101,102,103,104,105,106,107,108,109,110,111,112,113,114,115,116,117,118,119,120,121,122,123,124,125,126,127,128,129,130,131,132,133,134,135,136,137,138,139,140,141,142,143,144,145,146,147,148,149,150,151,152,153,154,155,156,157,158,159,160,161,162,163,164,165,166,167,168,169,170,171,172,173,174,175,176,177,178,179,180,181,182,183,184,185,186].

Chemical Exposure	Evidence of Breast or Mammary Carcinogenicity in Human or Rodent Studies ^a^	Evidence of Elevated ^b^ Exposure in Firefighters or Firefighter Environment	Exposure Sources/Events
Acetaldehyde ^c^	Rodent Female rats exposed to acetaldehyde had an increased incidence of benign mammary gland tumors (fibroma or fibroadenoma) [35]. Statistically significant differences in malignant mammary tumors were also observed in female rats [36,37]. Some tumors were observed in treated males [37].	Firefighting environment Area air levels during live fire training using OSB fuel were higher than applicable ceiling levels [38]. Average air sample concentrations during fire overhaul exceeded the NIOSH “lowest feasible concentration” [39].	Bushfire smoke [40], wildfires [41,42] and other biomass burnings [43,44]
			Structural or staged structural fires [39,45,46,47]
			Knockdown [45]
			Fire overhaul [39,45,48]
			Smoke from live fire trainings [49], including using OSB (oriented strand board) and pallet and straw as fuel [38]
Acrylonitrile	Rodent Both inhalation and ingestion [drinking water and stomach tube] have been shown to increase the incidence of mammary gland tumors in female rats [50,51,52]. Prenatal exposure followed by postnatal inhalation also induced mammary tumors in female rats [53].	NA	Vehicle fires [54]
			Biomass burnings [44]
Benzene ^c^	Human A large California study found a statistically significantly higher risk of the ER-/PR- breast tumor subtype with higher levels of ambient air levels of benzene [55]. A Polish study of women who worked with benzene had a non-significantly elevated risk of premenopausal breast cancer (OR: 0.84–2.80) [56], with a higher risk for those exposed 11–20 years before diagnosis. A 2012 report from the Institute of Medicine describes the association between benzene and breast cancer as suggestive, based on both epidemiologic and nonhuman data [57]. Occupational exposure to benzene was associated with increased male breast cancer in a 2018 study [58]. Rodent Oral administration caused mammary gland tumors (carcinomas and carcinosarcomas) in female mice [59,60]. Inhalation and ingestion by stomach tube increased the incidence of malignant mammary gland tumors in female rats [61,62].	Firefighters Exhaled breath concentrations of firefighters and instructors were statistically significantly higher after live fire training compared to before (using OSB or pallet and straw as fuel) [63] and in firefighters after controlled structure [64,65] and residential fires [66] compared to pre-exposure, even when SCBA was worn. A separate study found exhaled breath concentrations of benzene after controlled-structure burns were >2-fold pre-burn levels (non-significant; small sample size) [67]; these were statistically significantly correlated with off-gassing from PPE (personal protective equipment). Median urinary concentrations of benzene metabolites increased after firefighting [68] and following smoke exposure [69]. Firefighter instructors also had statistically significantly higher benzene urinary metabolite concentrations following fire training, with some concentrations exceeding ACGIH biological exposure indices [70]. Firefighter environment Median personal air concentrations for attack, search, and overhaul firefighters working in controlled residential fires were 40, 38, and 0.9 ppm, respectively, which are well above or close to the NIOSH STEL (short-term exposure limit) of 1ppm [71]. Median personal air concentrations for firefighters and instructors exceeded applicable short-term exposure limits during training fires [38]. In a study following NY firefighters, benzene was present at 12 of 14 fires monitored, all of which exceeded the NIOSH STEL of 1 ppm [72]. In an Australian study measuring atmospheric concentrations following simulated industrial fires, benzene concentrations exceeded the Australian 8-h time-weighted average exposure standards [73], including inside structural firefighting ensembles.	Smoke from fire training structures [49], including using OSB or pallet and straw as fuel [63]
			PPE off-gassing [67,74]
			Structural or staged structural fires [39,45,46,47,64,70,72,73,75,76,77,78,79] and compartment fire behavior training [80] and drills [68]
			Vehicle fire smoke [54,72]
			Knockdown [45]
			Overhaul [39,45,48]
			Diesel oil fire [81]
			Bushfire smoke [40], wildfires [82,83,84], and biomass burnings [43,44]
			Smoke diving stimulators [69]
1,3-butadiene ^c^	Human Women in rubber manufacturing plants with the highest-level exposure to styrene and butadiene had non-statistically significantly higher breast cancer mortality compared to women with no exposure [85]—due to the high correlation between the two chemicals, they were not analyzed separately. This was the only study available measuring occupational exposure to butadiene [23]. Rodent Inhalation exposure caused malignant and benign mammary tumors in rats and malignant mammary tumors in female mice [86,87].	NA	Live emergency fires and other live fire events [79,88], including municipal fires [also stimulated] [76,77], vehicle fires [54], and biomass burnings [43,44]
Dioxins and other dioxin-like compounds (including furans but excluding dioxin-like PCBs)	Human Women with higher blood levels of 2,3,7,8-tetrachlorodibenzodioxin (TCDD) who had lived near a dioxin accident in Seveso, Italy, in 1976 had 2–3 times the risk of developing breast cancer later in life, although this was not statistically significant [89]. Another study found an increased risk of breast cancer metastasis among patients with high BMI [90]. Positive associations have also been found between TCDD exposure from municipal solid waste incinerators and invasive breast cancer risk [91] and low-dose exposures and ER+ breast cancer (indicating a possible non-monotonic dose response) [92]. Overall, few other studies have investigated cancer in women associated with TCDD exposure. IARC has classified TCDD as a human carcinogen, based on all cancer sites combined, rather than for any specific site [93]. However, much of the evidence is based on male occupational cohort studies [89,93]. Rodent Dioxins have not been demonstrated to produce mammary tumors in standard experiments in adult rodents [23] and may instead reduce mammary tumors in mice, as evidenced by TCDD. However, in a 2-year gavage study in female rats with the dioxin-like compound, 2,3,4,7,8-pentachlorodibenzofuran, there were statistically significantly increased incidences of mammary gland carcinomas for two dose groups when compared to controls; these also exceeded the historical control range. There was a trend towards to lower adjusted incidences in higher dose groups [94]. Dioxins are known to bind to the aryl hydrocarbon receptor and may contribute to breast cancer by altering mammary gland development or hormone signaling pathways [23].	Firefighters Serum levels of several furans, including 2,3,4,7,8-pentachlorodibenzofuran, were statistically significantly higher pre- or post-controlled fire exposure compared to the general US population [95]. Serum congener levels of PCDD/Fs in Taiwanese fire scene investigators, although not firefighters, were also higher than those of the general Taiwanese population [96]. Serum levels of 1,2,3,4,6,7,8-heptaCDD (HpCDD), a dioxin congener, following structural firefighting exceeded both the US and Taiwanese general populations, although this study sample was small with only 12 participants [97]. A Russian study found statistically significantly elevated levels of HpCDD in former and current firefighters compared to non-firefighters, with chemical levels declining as the years since employed as a firefighter increased [98].	Structural firefighting [97] including controlled residential fires [95] and simulated burns [99,100]
Polybrominated diphenyl ether (PBDE) flame retardants	Human A study of Alaskan Native women had a non-significant increased risk of breast cancer with higher levels of PBDE-47 [101]. Adipose tissue levels of several PBDE congeners were associated with higher odds of breast cancer in Chinese women [102]; however, a California-based study found no statistically significant associations [103]. Rodent PBDEs do not seem to have been tested in rodents for mammary gland tumor induction [23,104]. However, PBDE mixtures have been shown to increase cell proliferation of estrogen-sensitive breast cancer cells [105,106] and to alter mammary gland development in rats exposure during and after gestation [107].	Firefighters PBDE metabolites in serum were higher in firefighters compared to general US population [97,108], with one study reporting levels 2- to 3-fold higher. Levels of BDE-209 were statistically significantly higher in firefighters before and after fire exposure compared to the general population [95]. Median PBDE levels in serum were statistically significantly higher in Korean firefighters compared to the Korean general population [109]; concentrations were correlated with length of service and years dedicated to on-site dispatch work. Firefighter environment Median levels of PBDEs in fire station dust [110,111] and air [111] were elevated compared to houses and other workplaces. Using silicone wristband measurements, exposures for on-duty firefighters not responding to a fire were generally higher than for off-duty [112].	Fire station dust [110,111] and air [111]
			Controlled residential fires [95]
			Used/soiled PPE [113,114,115], including off-gassing of uniforms stored in private vehicles [116]
			Unused PPE, including on hoods and gloves [114]
			Cross-contamination during PPE laundering [117]
Replacement flame retardants: non-PBDE brominated flame retardants (BFRs) and organophosphorus flame retardants (OPFRs)	Rodent The BFR 2,2-bis(bromomethyl)-1,3-propanediol administered as a commercial mixture increased the incidence of mammary gland tumors in rats of both sexes [118]. A metabolite of tris (2,3-dibromopropyl) phosphate (TDBPP; an OPFR), 2,3-dibromo-1-propanol, produced mammary gland adenocarcinomas in female rats [32]. 3-monochloropropane-1,2-diol (3-MCPD), an indirect metabolite of tris(1,3-dichloro-2-propyl)phosphate, which is structurally similar to TDBPP and more commonly used today, also produced mammary tumors in female rats [33].	BFRs—Firefighter environment Concentrations of 8 of 11 NBFRs measured were statistically significantly higher in fire station dust samples compared to residential dust samples [119], including 4 that were direct replacements for PBDEs. Using silicone wristband measurements, exposures for on-duty firefighters were generally higher than for off-duty [112]. OPFRs—Firefighters Compared to female office workers and women in NHANES, female firefighters had higher concentrations of OPFRs or their metabolites, with the greatest difference in median levels for DBuP, BDCPP, BCEP [120]. Compared to office workers, female firefighters also had higher detection frequencies [120]. Another study found median urinary metabolite concentrations, including BCPP, BDCPP, and BCEtP, to be higher in firefighters compared to the general population [121]. Several OPFR metabolites were higher post-fire compared to pre-fire [95], with DPhP increasing statistically significantly. BDCPP and DPhP were also statistically significantly higher after fire exposure when compared to the general population [95]. OPFRs—Fire station Median levels of OPFRs in fire station dust [111] and air [111] were elevated compared to houses and other workplaces.	Fire station dust [111,119] and air [111]
			Structural firefighting, including controlled residential fires [95,121]
			Used/soiled and unused PPE [95,115], including off-gassing of uniforms stored in private vehicles [116]
			Cross-contamination during PPE laundering [117]
Isoprene ^c^	Rodent In rats of both sexes, exposure by inhalation caused mammary gland fibroadenomas [50,122,123]. In male rats, exposure also caused mammary gland carcinomas [123].	NA	Structural firefighting [124]
			Soiled PPE, including turnout jacket and gloves [124]
Per- and polyfluoroalkyl substances [PFASs]	Human A study that measured PFAS exposures during pregnancy found statistically significantly increased breast cancer risk (in the mothers) with higher PFOSA exposures [125]. This study found no statistically significant associations for PFOA. A more recent study reported an association between PFOS and ER+/PR+ breast cancer and between low levels of PFOA and PFOS and ER−/PR− breast cancer [126]. A study on Inuit women in Greenland reported statistically significantly elevated levels of PFOS and PFOA among breast cancer control cases, but since this population is exposed to a number of other breast-cancer-relevant chemicals, it is difficult to attribute risk to individual compounds [23,127]. The National Academies of Sciences concluded there to be limited suggestive evidence of the association between PFASs and breast cancer [128] Of six more recently published studies, four studies demonstrated an increased risk of breast cancer associated with PFASs (Supplemental Table S5). Rodent Perfluorooctanoic acid (PFOA) exposure alters the development of the mammary gland [129,130]. Effects on the developing mammary gland were observed at lower doses than other effects [131,132], and one rodent cancer study showed equivocal evidence of increased mammary gland tumors [133]. A study with adult rats found statistically significantly increased risk of benign mammary tumors with low doses of PFOS in the diet, but a statistically significantly decreased risk in the high-dose group [134].	Firefighters In Finnish firefighters, serum concentrations of PFHxS and PFNA increased after three consecutive training sessions using firefighter foam [135]. Compared to office workers, firefighters had higher geometric mean concentrations of PFASs, including PFHxS, PFUnDA, PFNA [136]. Serum levels of PFDoA, PFNA, and PFSA were found to be higher in a group of US volunteer firefighters compared to the general population, with levels of PFDA and PFDoA positively associated with years of firefighting [137]. PFDA levels were found to be 3 times higher in a group of California firefighters compared to NANES adult males [138]. Firefighters—PFOA Serum levels were 2-fold higher compared to US general population levels [97]. Compared to firefighters whose turnout gear had been professionally cleaned within a year, firefighters who had not had their gear cleaned had statistically significantly higher concentrations [138]. Firefighters—PFOSA/PFOS Former firefighter employment statistically significantly correlated with higher PFOS serum concentrations [139]. Serum levels were higher in firefighters than in those who reported other employment or no employment, but only reached significance compared to the latter [140]. This study only had 37 firefighters. In a group of Australian firefighters, serum levels of PFOS were 6–10 times higher compared to the general Australian and Canadian populations [141]. Blood donation was negatively correlated to PFOS and PFOA levels, while years of a job with AFFF contact were positively associated [141]. Firefighting environment—PFOA Using silicone wristbands, statistically significantly higher concentrations were observed when firefighters were on duty and responding to a fire [112].	Firefighting foam [135,136,138,141]
			Fire station dust [142], particularly in apparatus bays and turnout gear locker rooms
			Used turnout gear [142]
			New turnout gear, including in the outer layer, moisture barrier and thermal layers [143]
Polycyclic aromatic hydrocarbons [PAHs] ^c^	Human There is some evidence of increased post-menopausal breast cancer risk among women with occupational PAH exposures [144]. Women with PAH-DNA adducts and variants in genes involved in gene repair, tumor suppression, and PAH metabolism had increased risks of breast cancer [145,146,147,148,149]. A more recent study found that among women with an underlying genetic susceptibility to breast cancer, higher plasma PAH-albumin adduct levels were associated with 2–3 times greater breast cancer risk when compared to non-detectable levels [150]. Rodent Seven PAHs (benzo[a]pyrene, 3-methylcholanthrene, 7,12-dimethylbenz[a]anthracene, dibenzo[a,l]pyrene, dibenzo[a,i]pyrene, dibenzo[a,h]pyrene, dibenz[a,h]anthracene) and six nitro-PAHs (1,3-dinitropyrene, 1,8-dinitropyrene, 1-nitropyrene, 2-nitrofluorene, 4-nitropyrene, 6-nitrochrysene) increased the risk of mammary gland tumors in animal models [26].	Firefighters Pre- vs. post-exposure: urinary metabolites Compared to pre-exposure, firefighters often had statistically significantly increased concentrations of urinary PAH metabolites after exposure to structural or controlled residential fires [66,151], wildland firefighting [152,153], on-shift fire suppression [154], and other work shifts with firefighting-related emergency calls [109,152]. One study found that OH-PAH levels were also associated with firefighters’ exposure duration, age, length of service, and years dedicated to on-site dispatch [109]. Firefighters (and/or instructors) also had higher OH-PAH levels following live fire training sessions, including at burn houses [69,155], and stimulated compartment fires [69,156,157], and using various different fuel types [63,69,157] (e.g., OSB, particleboard, and conifer plywood). Pre- vs. post-exposure: skin deposition Compared to pre-exposure, skin loadings for total PAHs were statistically significantly increased by an average of 4-fold after exposure to wood smoke [155]. PAH levels were also statistically significantly higher on the skin after emergency fire suppression [158] and smoke diving exercises [159], and on the neck following the suppression of controlled structure fires [64]. Elevated levels of benzo(a)pyrene and 3-methylcholanthrene were found on the skin (neck or hands) following a fire training exercise [160] Firefighters vs. general population or controls Many of the OH-PAHs found pre-training and pre-firefighting were above general non-smoking population medians [63,66]. A Korean study found that serum levels of PAHs were statistically significantly higher in firefighters compared to the Korean general population [109]. Pre-shift and post-shift median concentrations of OH-PAHs were also higher among wildland firefighters than among the general population [152]. Compared to control subjects, firefighters who engaged in fire combat activities had statistically significantly higher concentrations of urinary PAH metabolites (up to 340%) [161]. Firefighter environments Median personal air concentrations for firefighters and instructors exceeded applicable short-term exposure limits during training fires [38], with naphthalene being responsible for 66–68% of the total PAH concentration depending on the fuel package. Personal air concentration sampling for attack and search firefighters working in controlled residential fires found median concentrations of 23,800 and 17,800 µg/m ^c^, respectively, which are well above the 1000 µg/m ^c^ ACGIH excursion limit for coal tar pitch volatiles [71]. Area air concentrations measured from a modern living room during a fire period have also been found to be above the ACGIH excursion limit, with 57% of total PAHs being IARC-classified as probably or possibly carcinogenic and 2% as known carcinogenic [71]. Median concentrations in fire station dust and air samples were substantially higher than those found in dust in homes [110,111]. Firefighters who responded to an active fire were exposed to statistically significantly higher concentrations of lower-molecular-weight PAHs than those who did not [162]. Using silicone wristband measurements, exposures for on-duty firefighters were generally higher than for off-duty (particularly when a fire was involved) [112], as well as for shifts with fire vs. shifts with no fire [112,163]. In an Australian study measuring air concentrations in a stimulated industrial fire, PAH concentrations inside the structural firefighting ensembles approached or exceeded the Australian 8-hr time-weighted average total PAH concentrations for a range of industrial work environments such as coke ovens and tar distillation (no standard currently exists for firefighters) [73].	Live emergency fires [88,154,158]
			Structural or staged structural fires [39,45,46,64,66,73,75,100,151,163,164]
			Live fire trainings [38,49,81,155,156,157,165,166,167,168,169] and smoke diving exercises [69,159]
			Dust and air in fire stations [110,111,170,171]
			Vehicle bays and fire truck cabs [158]
			Fire engines (console and bodyguard) [160]
			Soiled PPE [73,74,113,115,158,160,162,168,172], including off-gassing of uniforms stored in private vehicles [116]
			incident command post [173]
			Overhaul [39]
			Knockdown [45]
			Overhaul [39,45,174]
			Wildfires, prescribed burns and other biomass burnings [42,43,44,152,163,167,175,176]
Polychlorinated biphenyls [PCBs] ^c^	Human IARC considers there to be limited evidence of the association between PCBs and breast cancer due to biological plausibility but inconsistent associations in studies [177,178]. Rodgers et al. attribute these inconsistent associations to be due to the lack of consistency in the congener types analyzed [23]. Levels of several PCB congeners found in adipose tissue were associated with higher odds of breast cancer in Chinese women [179]. Recent studies of PCB exposure and breast cancer have generally shown positive associations (Supplemental Table S3). Rodent The PCB mixture Aroclor 1254 statistically significantly increased the incidence of mammary gland fibroadenomas in female rats at the middle dose, with a non-significant increase at the low dose [178]. However, the high dose resulted in a statistically significantly decreased incidence of spontaneous mammary gland tumors [180]. Aroclor 1260 also repressed mammary gland tumor incidence [180]. The PCB metabolite 4’OH-PCB-61 increased mammary gland carcinoma incidence at the lowest administered dose in a female mouse strain known for its low incidence of mammary tumors [178,181]. Mechanistic PCBs include 209 different congeners with different biological activities: estrogenic, tumor promotion, induction of metabolizing enzymes (CYPs), oxidative damage [178]. Some PCBs also exhibit dioxin-like activity and bind to the aryl hydrocarbon receptor.	Total PCBs (TEQ and body burden) were higher in current fire firefighters compared to former or non-firefighters [98]. Current and former firefighters had statistically significantly higher levels of PCB-114, PCB-156, BCP-157, and PCB-167 compared to non-firefighters; PCB-105 and PCB-118 were nearly statistically significantly lower in non-firefighters [98]. A study by Park et al. (2015), however, reported lower median serum PCB concentrations among Californian firefighters when compared to the general US population [108].	Dust found in fire stations [110]
			Simulated house fires [100]
			Soiled turnout gear [113]
Styrene ^c^	Human See epidemiological evidence from 1,3-butadiene. A more recent large longitudinal cohort of women exhibited a non-significant, suggestive association between living in an area with high air concentrations of styrene and breast cancer (both ER+ and in general) [182]. Rodent Exposure to styrene in drinking water increased the incidence of mammary fibroadenomas in female rats [183]. Inhalation studies have reported mixed findings in the same strain of rats, with one study reporting a statistically significant increase in malignant tumors and in malignant and benign tumors combined (with positive trends), and the other a dose-related decrease in adenocarcinomas [183,184].	Exhaled breath concentrations of firefighters and instructors statistically significantly elevated after live fire training compared to before (using OSB or pallet and straw as fuel) even with SCBA worn [63]. Exhaled breath concentrations after controlled structure burns were >2-fold pre-burn levels (non-significant; small sample size) and statistically significantly correlated with off-gassing from firefighters’ used PPE [67]. Following controlled residential and training fire responses, the urinary metabolite of styrene (MADA) was statistically significantly increased in firefighters and instructors [70], with median levels during the training session exceeding the smoking general population levels.	Training fires [70], including using OSB or pallet and straw as fuel [63]
			off-gassing from used PPE [67,74]
			Vehicle fire smoke [54]
			Diesel oil fires [81]
			Biomass burnings [43,44]
			Knockdown [45]
			Structural fires (including stimulated) [45,46,70,73,75,76,77,79]

^a^ Evidence of breast or mammary carcinogenicity in humans is compiled from Rodgers et al. 2018 [23] and Brody et al. 2007 [185], and evidence from rodent studies is compiled from Kay et al. 2024 [26] and Rudel et al. 2007 [186]. ^b^ Cases of elevated exposure include higher exposure in firefighters compared to the general population or other occupations, higher exposure post-firefighting event compared to pre-event, and evidence of exposure exceeding occupational safety limits in the environment. ^c^ Chemical is classified as carcinogenic, probably carcinogenic, or possibly carcinogenic to humans by the International Agency for Research on Cancer.

## 4. Discussion

The current study identified twelve chemical exposures that were elevated among firefighters and also associated with increased breast cancer risk in epidemiological studies or increased incidence of mammary gland tumors in rodents (Table 1 and Table 2). We found strong evidence of elevated firefighter exposure for nine of these chemicals or chemical groups: benzene, PAHs, acetaldehyde, styrene, dioxin-like compounds, PBDEs, replacement flame retardants, PFASs, and PCBs. Six of these exposures were higher in firefighters compared to the general population (dioxins, PBDEs, replacement flame retardants, PFASs, PAHs, PCBs); a different set of six were higher after firefighting activity compared to before firefighting activity (benzene, dioxins, replacement flame retardants, PFASs, PAHs, styrene), and five were present at elevated concentrations in firefighting-related environments compared to non-firefighter work environments or exceeded occupational exposure limits (acetaldehyde, benzene, PBDEs, replacement flame retardants, PFASs, PAHs). Two of these firefighter exposures—benzene and PAHs—had strong evidence of increased breast cancer risk, while six had moderate evidence, and four had limited evidence of increased risk.

To the best of our knowledge, this is the first study to integrate exposure, toxicology, and epidemiological evidence to assess the potential increased risk of breast cancer among firefighters. Evidence of increased risk in firefighters due to specific chemical exposures is difficult to obtain, so our study integrated data about firefighter exposures and the potential for these exposures to increase breast cancer risk based on epidemiological studies in other populations, rodent cancer studies, or both. This approach is consistent with that of IARC and other authorities that rely on evidence from rodent studies or on epidemiology in a specific population, such as workers, to predict that an exposure is likely to cause cancer in humans more generally. These findings provide a basis for recognizing these risks and taking action to reduce exposures.

Exposure to breast-cancer-relevant chemicals was reported to occur through various events and from various sources, including structural fires, live fire training scenarios, biomass burnings and wildland fires, vehicle fires, off-gassing from PPE, and fire station dust and air. Most of these events and sources are linked to multiple co-occurring breast-cancer-relevant chemicals. For example, many of these chemicals are present in petroleum or diesel fuel and exhaust which firefighters are exposed to, including benzene, butadiene, and PAHs. Emissions from firefighter trucks may be an important source of these exposure that could be mitigated, for example, by modifications to truck bay zones where fuel exhaust contaminants are elevated compared to other fire station areas [158,170,174].

In addition to chemicals linked to fuel exhaust, various other chemicals have been found in fire station dust or air, including PBDEs, replacement flame retardants, and PCBs. In some cases, measured ambient concentrations were statistically significantly higher than levels found in homes and other workplaces, suggesting that that the fire station is a potential source of exposure that can be modified (Table 2). Chemicals found in fire station dust and air may be due not only to the fuel and combustion emissions previously mentioned, but also to the off-gassing of chemicals from equipment or soiled PPE [67].

Unsurprisingly, many of these chemicals, most notably benzene and PAHs, are found in combustion products that firefighters commonly encounter in live fire scenes. Although firefighters often wear PPE to minimize their exposure to the high levels found at fire scenes, exposure was reported to occur regardless of wear in some instances. A study on firefighters performing suppression and overhaul of controlled structure fires reported statistically significantly elevated concentrations of PAHs on the neck, and changes in urinary PAH metabolite levels were statistically significantly correlated with personal air PAH concentrations [64]. The study authors hypothesized that even when full protective equipment was worn, PAHs may have been dermally absorbed through the neck due to a lower level of dermal protection afforded by the particulate-blocking hoods [64]. Dermal exposure has been proposed as a probable exposure pathway by the investigators of studies showing elevated exposure levels in firefighters even when a self-contained breathing apparatus (SCBA) is worn [64,70].

Furthermore, while an SCBA is often worn to reduce respiratory exposure, it is not worn in all instances when exposure may occur. For example, firefighters may opt not to wear an SCBA when conducting overhaul and outside vent assignments because it is poorly suited to the work tasks [174], when wildland firefighting where it may be impractical [176], or during vehicular fire suppression [54]. These activities are potential sources of elevated exposure in firefighters (Table 2). As previously noted, firefighters may also be exposed in the fire stations or when doffing PPE that may off-gas [67], both cases when firefighters are not expected to be wearing protective equipment.

Another important potential source of exposure is from carcinogenic flame retardants used in firefighter gear. Trowbridge et al. (2021) found urine levels of several OPFR metabolites in firefighters that were detected more frequently and at higher levels than in a comparison group of office workers, with median levels of BDCPP—the OPFR detected at the highest levels—being 5 times higher in firefighters than in office workers [120]. Several OPFRs or their degradation products and impurities have been shown to be carcinogenic in rodent studies at multiple sites including the mammary gland [25]. While firefighters need gear that will protect them from fire, these elevated exposures and associated risks suggest a need to develop alternative approaches to shift away from using these carcinogenic flame retardants.

Previous studies have shown that firefighters’ exposures are reduced by field decontamination of PPE, routine laundering of PPE, and the use of skin cleansing wipes, handwashing, or showering post-exposure. Unfortunately, these activities often do not lead to complete decontamination, and effectiveness may vary depending on the method used. For example, field decontamination using a wet-soap method on turnout jackets led to a median reduction in PAH levels of 85%, while using a dry-brush method resulted in a median reduction of 23% [187]. Routine laundering of firefighter hoods has been shown to reduce residual levels of OPFRs, non-PBDE flame retardants (NPBFRs), and PAHs [117]. However, this same study showed that cross contamination of flame retardants can occur during laundering as PBDE levels were on average 43% higher after laundering, and washing previously unexposed hoods with heavily exposed hoods also led to higher levels of PBDEs, NPBFRs, and OPFRs in the unexposed hoods [117]. This suggests that laundering effectiveness depends on the type of chemical in the material and the level of contamination present in the laundering load [117]. Using skin cleaning wipes has also been shown to reduce contamination levels, reducing PAH contamination on the neck by a median of 54% [187]. Preliminary results suggest that dermal absorption of volatile compounds may be reduced by unzipping and airing out turnout gear after fire exposure [188].

Broader systemic change is also necessary to supplement individual action. Manufacturers can avoid using carcinogenic and toxic chemicals as flame retardants or as fabric treatment in firefighting gear. Federal and state governments can also be proactive. Several state governments have passed laws restricting or banning PFAS-based firefighting foams [189] and requiring reporting of the presence of PFASs in firefighting gear [190]. The US Congress has also made the decision to phase out the use of PFASs in military firefighting foams by 2024 [191].

Due to the nature of the firefighting occupation, some exposures may not be able to be eliminated. For this reason, local, state, and federal governments can include breast cancer in their firefighter cancer presumption laws (which presume that certain cancers are work-related) or entitle workers to compensation benefits. While current presumption laws in many US states consider firefighters’ claims of their cancer as an occupational disease, some states may not cover breast cancer or are more likely to challenge a firefighter’s breast cancer claim, making it harder to receive benefits [192]. To ease firefighters’ compensation claims, or to support claims, it is encouraged that firefighters document their exposures and be aware of the breast-cancer-related exposures on the job, such as those identified in this manuscript. Mobile applications that are designed to help firefighters track their exposures may facilitate this process [193].

### 4.1. Limitations

There are limitations associated with our study. Because chemical effects on breast carcinogenicity remain largely understudied, the list of potential mammary gland carcinogens we used to identify firefighter exposures is likely to represent only a subset of the exposures that may increase breast cancer risk. Relevant data from epidemiologic studies of these exposures and breast cancer are not available or are sparse, which has the effect of limiting the number of chemicals with stronger evidence of breast cancer associations. For example, we classified some firefighter exposures such as 1,3-butadiene as having moderate evidence for an association with breast cancer, but that may understate the potential association with breast cancer. Butadiene is also present in air pollution, and associations between air pollution and cancer risk are well established [194], with mounting evidence for associations with breast cancer, including in women with high inherited breast cancer risk [195].

We also did not evaluate chemicals that may increase susceptibility to breast cancer by altering mammary gland development or disrupting hormonal processes [196]. Additionally, chemicals may act additively or synergistically with other chemicals to increase the risk of breast cancer beyond their individual risks [197,198]; however, current data on this are limited. While our study highlighted chemicals for which firefighters had elevated exposures at a single point in time, we did not assess the relationship with duration of exposure, low chronic exposures, and acute peak exposures that may all be important in the development of breast cancer.

Finally, we may have missed some important firefighter exposures because some of the occupational safety levels that we used as criteria for elevated exposure in articles we reviewed may be out of date or unprotective. In either case, exposures below these occupational safety levels may still increase risk. On the other hand, elevated levels measured at the fire scene may be mitigated by PPE, although as previously mentioned, PPE is not always effective, and firefighters may not wear PPE in all situations.

### 4.2. Conclusions and Recommendations

Our study clarifies the evidence that at least a dozen firefighter chemical exposures may be increasing the risk of breast cancer in the firefighting occupation. Since females have been a small proportion of firefighters in the past, breast cancer has rarely been noted as an occupational hazard; this pattern has changed with many more females entering the fire services. Thus, the information compiled here is vital for informing efforts to further reduce exposures to the twelve chemicals or chemical groups identified as high priorities based on our study.

We present several recommendations based on our findings. First, because of the burden of chemical exposures on firefighters due to the nature of their occupation, we encourage limiting toxic chemicals in the manufacture of firefighting equipment, for example, firefighting foams and turnout gear. Second, although decontamination procedures may not eliminate exposure to all toxic chemicals, they are still effective in reducing exposures. Third, laws designed to compensate firefighters for work-related diseases should recognize that breast cancer is a likely occupational disease for firefighters. There are also areas where additional research may help better understand risks. For example, research aiming to uncover potential breast carcinogenic exposures among female firefighters, including non-targeted analyses, may identify additional risks that could be mitigated. Additional studies of the association between the twelve high-priority exposures presented in this paper and the risk of breast cancer in firefighters may clarify associations and risks. In addition, we recommend additional studies on the association between the firefighting occupation and health outcomes more common in women, such as breast and ovarian cancer, infertility, endometriosis, and uterine fibroids. Finally, firefighters who are pregnant or breastfeeding seek guidance on exposure reduction that will protect their offspring, and additional research and guidance in this area are needed.

## Figures and Tables

**Table 1:** List of breast-cancer-relevant chemical or chemical groups included in firefighter exposure studies along with the number of firefighter exposure studies that included chemical in the study and assessment classifications for the evidence of elevated exposure in firefighting and likelihood of breast cancer following adult exposures.

Chemical or Chemical Group	Number of Articles Including Chemical in Study	Evidence of Elevated Exposure in Firefighting	Likelihood of Breast Cancer Following Adult Exposures
Benzene	43	Strong	Probable
Polycyclic aromatic hydrocarbons [PAHs]	75	Strong	Probable
Acetaldehyde	20	Strong	Moderate
Styrene	24	Strong	Moderate
Replacement flame retardants (halogenated OPFRs and BFRs)	9	Strong	Presumed moderate ^a^
PBDE flame retardants	13	Strong	Limited
Dioxins and other dioxin-like compounds	14	Strong	Limited
Per- and polyfluoroalkyl substances [PFASs]	11	Strong	Limited
Polychlorinated biphenyls [PCBs]	11	Strong	Limited
Acrylonitrile	2	Some	Moderate
1,3-butadiene	10	Some	Moderate
Isoprene	5	Some	Moderate
Carbon tetrachloride	1	Inadequate evidence	NA
1,2-Dibromoethane	2	Inadequate evidence	NA
1,1-Dichloroethane	1	Inadequate evidence	NA
1,1-Dichloroethylene	1	Inadequate evidence	NA
1,2-Dichloroethane	1	Inadequate evidence	NA
Dichloromethane	6	Inadequate evidence	NA
1,2-Dichloropropane	1	Inadequate evidence	NA
1,4-Dioxane	2	Inadequate evidence	NA
Ethanol	5	Inadequate evidence	NA
Isoeugenol	1	Inadequate evidence	NA
Organochlorine pesticides	2	Inadequate evidence	NA
Organophosphate pesticides	1	Inadequate evidence	NA
Perchloroethylene [tetrachloroethylene/PCE]	7	Inadequate evidence	NA
Toluene diisocyanates	1	Inadequate evidence	NA
Trichloroethylene [TCE]	4	Inadequate evidence	NA
Vinyl chloride	1	Inadequate evidence	NA

^a^ Rodent mammary tumors observed for 2,3-dibromo-1-propanol, 3-monochloropropane-1,2-diol, and 2,2-bis(bromomethyl)-1,3-propanediol. All three of these are short-chain halogenated alkyl alcohols. Other OPFRs and BFRs have either not shown mammary tumors or not been tested.

## Data Availability

The original contributions presented in the study are included in the article/Supplementary Material; further inquiries can be directed to the corresponding author.

## Supplementary Materials

The following supporting information can be downloaded at: https://www.mdpi.com/article/10.3390/toxics12100707/s1, Table S1: List of 294 a priori breast cancer relevant chemicals compiled from Kay et al. (2024) and Rodgers et al. (2018). Column C indicates whether chemical induced mammary gland tumors in rodents or is linked to breast cancer in epidemiological studies; Table S2: Search terms and number of studies identified for select breast carcinogens published between June 2016 and June 2022; Table S3: Articles on select chemicals reviewed for epidemiological evidence of breast cancer along with summary of findings; Table S4: List of 682 articles identified through PubMed in initial search using the keywords “exposure” and “firefighter” and published between January 2005 and May 2022. List includes the article PMID, abstract, title, author, and journal. These PMIDs were uploaded to AbstrackR and inclusion/exclusion critera were applied to abstract and title; Table S5: List of 129 articles relevant to firefighter exposures and reviewed for mention of breast cancer relevant chemicals. Column A indicates whether article was included because it was identified through PMID and met the inclusion exclusion/critera [PubMed] or whether it was identified through IARC Monograph 98 [IARC]; Table S6: Articles on firefighter exposures that mentioned breast cancer relevant chemicals. 110 unique articles listed. Column A is the article [starting with the PMID article or author name (if no PMID is available) and ending with year article was published].

## References

[1] Daniels R.D., Kubale T.L., Yiin J.H., Dahm M.M., Hales T.R., Baris D., Zahm S.H., Beaumont J.J., Waters K.M., Pinkerton L.E. (2014). Mortality and cancer incidence in a pooled cohort of US firefighters from San Francisco, Chicago and Philadelphia (1950–2009). Occup. Environ. Med..

[2] LeMasters G.K., Genaidy A.M., Succop P., Deddens J., Sobeih T., Barriera-Viruet H., Dunning K., Lockey J. (2006). Cancer risk among firefighters: A review and meta-analysis of 32 studies. J. Occup. Environ. Med..

[3] Ma F., Fleming L.E., Lee D.J., Trapido E., Gerace T.A. (2006). Cancer incidence in Florida professional firefighters, 1981 to 1999. J. Occup. Environ. Med..

[4] Glass D.C., Pircher S., Del Monaco A., Hoorn S.V., Sim M.R. (2016). Mortality and cancer incidence in a cohort of male paid Australian firefighters. Occup. Environ. Med..

[5] Tsai R.J., Luckhaupt S.E., Schumacher P., Cress R.D., Deapen D.M., Calvert G.M. (2015). Risk of cancer among firefighters in California, 1988–2007. Am. J. Ind. Med..

[6] Marjerrison N., Jakobsen J., Grimsrud T.K., Hansen J., Martinsen J.I., Nordby K.C., Veierød M.B., Kjaerheim K. (2022). Cancer incidence in sites potentially related to occupational exposures: 58 years of follow-up of firefighters in the Norwegian Fire Departments Cohort. Scand. J. Work Environ. Health.

[7] Sritharan J., Kirkham T.L., MacLeod J., Marjerrison N., Lau A., Dakouo M., Logar-Henderson C., Norzin T., DeBono N.L., Demers P.A. (2022). Cancer risk among firefighters and police in the Ontario workforce. Occup. Environ. Med..

[8] Soteriades E.S., Kim J., Christophi C.A., Kales S.N. (2019). Cancer Incidence and Mortality in Firefighters: A State-of-the-Art Review and Meta-Analysis. Asian Pac. J. Cancer Prev..

[9] Ma F., Fleming L.E., Lee D.J., Trapido E., Gerace T.A., Lai H., Lai S. (2005). Mortality in Florida professional firefighters, 1972 to 1999. Am. J. Ind. Med..

[10] Pinkerton L., Bertke S.J., Yiin J., Dahm M., Kubale T., Hales T., Purdue M., Beaumont J.J., Daniels R. (2020). Mortality in a cohort of US firefighters from San Francisco, Chicago and Philadelphia: An update. Occup. Environ. Med..

[11] Muegge C.M., Zollinger T.W., Song Y., Wessel J., Monahan P.O., Moffatt S.M. (2018). Excess mortality among Indiana firefighters, 1985–2013. Am. J. Ind. Med..

[12] Daniels R.D., Bertke S., Dahm M.M., Yiin J.H., Kubale T.L., Hales T.R., Baris D., Zahm S.H., Beaumont J.J., Waters K.M. (2015). Exposure-response relationships for select cancer and non-cancer health outcomes in a cohort of U.S. firefighters from San Francisco, Chicago and Philadelphia (1950–2009). Occup. Environ. Med..

[13] Demers P.A., DeMarini D.M., Fent K.W., Glass D.C., Hansen J., Adetona O., Andersen M.H., Freeman L.E.B., Caban-Martinez A.J., Daniels R.D. (2022). Carcinogenicity of occupational exposure as a firefighter. Lancet Oncol..

[14] Pukkala E., Martinsen J.I., Weiderpass E., Kjaerheim K., Lynge E., Tryggvadottir L., Sparén P., Demers P.A. (2014). Cancer incidence among firefighters: 45 years of follow-up in five Nordic countries. Occup. Environ. Med..

[15] Glass D.C., Del Monaco A., Pircher S., Vander Hoorn S., Sim M.R. (2019). Mortality and cancer incidence among female Australian firefighters. Occup. Environ. Med..

[16] Hulett D.M., Bendick M., Thomas S.Y., Moccio F. (2008). A National Report Card on Women in Firefighting.

[17] Jahnke S.A., Haddock C.K., Jitnarin N., Kaipust C.M., Hollerbach B.S., Poston W.S.C. (2019). The Prevalence and Health Impacts of Frequent Work Discrimination and Harassment among Women Firefighters in the US Fire Service. BioMed Res. Int..

[18] Ly D., Forman D., Ferlay J., Brinton L.A., Cook M.B. (2013). An international comparison of male and female breast cancer incidence rates. Int. J. Cancer.

[19] CDC (2020). Male Breast Cancer Incidence and Mortality, United States—2013–2017.

[20] Chowdhury R., Shah D., Payal A.R. (2017). Healthy Worker Effect Phenomenon: Revisited with Emphasis on Statistical Methods—A Review. Indian J. Occup. Environ. Med..

[21] IARC (2010). Monographs on the Evaluation of Carcinogenic Risks to Humans, Volume 98: Painting, Firefighting and Shiftwork.

[22] IARC (2023). IARC Monographs: Occupational Exposure as a Firefighter.

[23] Rodgers K.M., Udesky J.O., Rudel R.A., Brody J.G. (2018). Environmental chemicals and breast cancer: An updated review of epidemiological literature informed by biological mechanisms. Environ. Res..

[24] Rudel R.A., Fenton S.E., Ackerman J.M., Euling S.Y., Makris S.L. (2011). Environmental exposures and mammary gland development: State of the science, public health implications, and research recommendations. Environ. Health Perspect..

[25] Rudel R.A., Ackerman J.M., Attfield K.R., Brody J.G. (2014). New Exposure Biomarkers as Tools For Breast Cancer Epidemiology, Biomonitoring, and Prevention: A Systematic Approach Based on Animal Evidence. Environ. Health Perspect..

[26] Kay J.E., Brody J.G., Schwarzman M., Rudel R.A. (2024). Application of the Key Characteristics Framework to Identify Potential Breast Carcinogens Using Publicly Available in Vivo, in Vitro, and in Silico Data. Environ. Health Perspect..

[27] Kerber S. (2012). Analysis of Changing Residential Fire Dynamics and Its Implications on Firefighter Operational Timeframes. Fire Technol..

[28] Ward E.M., Sherman R.L., Henley S.J., Jemal A., Siegel D.A., Feuer E.J., Firth A.U., Kohler B.A., Scott S., Ma J. (2019). Annual Report to the Nation on the Status of Cancer, Featuring Cancer in Men and Women Age 20–49 Years. J. Natl. Cancer Inst..

[29] Grant M.J., Booth A. (2009). A typology of reviews: An analysis of 14 review types and associated methodologies. Health Inf. Libr. J..

[30] Fahy R., Evarts B., Stein G.P. (2022). US Fire Department Profile 2020.

[31] Wallace B.C., Small K., Brodley C.E., Lau J., Trikalinos T.A. (2012). Deploying an interactive machine learning system in an evidence-based practice center: Abstrackr. Proceedings of the 2nd ACM SIGHIT International Health Informatics Symposium.

[32] NTP (2021). 2,3-Dibromo-1-Propanol.

[33] Faust J.B., August L.M., OEHHA (2011). Evidence on the Carcinogenicity of Tris(1,3-Dichloro-2-Propyl) Phoshate.

[34] IARC (2023). Agents Classified by the IARC Monographs.

[35] NTP (2021). Acetaldehyde.

[36] IARC (2010). Monographs on the Evaluation of Carcinogenic Risks to Humans, Volume 96: Alcohol Consumption and Ethyl Carbamate.

[37] Soffritti M., Belpoggi F., Lambertin L., Lauriola M., Padovani M., Maltoni C. (2002). Results of long-term experimental studies on the carcinogenicity of formaldehyde and acetaldehyde in rats. Ann. N. Y. Acad. Sci..

[38] Fent K.W., Mayer A., Bertke S., Kerber S., Smith D., Horn G.P. (2019). Understanding airborne contaminants produced by different fuel packages during training fires. J. Occup. Environ. Hyg..

[39] Bolstad-Johnson D.M., Burgess J.L., Crutchfield C.D., Storment S., Gerkin R., Wilson J.R. (2000). Characterization of firefighter exposures during fire overhaul. Aihaj.

[40] Reisen F., Brown S.K. (2009). Australian firefighters’ exposure to air toxics during bushfire burns of autumn 2005 and 2006. Environ. Int..

[41] Kelly J. (1991). Health Hazard Evaluation Report No. HETA 91–312–2185: Firefighter’s Exposure to Smoke during Fire Supppression Activities at Wildland Fires.

[42] US Department of the Interior National Park Service, NIOSH (1992). Health Hazard Evaluation Report No. HETA 90–0365–2415.

[43] Andreae M.O., Merlet P. (2001). Emission of trace gases and aerosols from biomass burning. Glob. Biogeochem. Cycles.

[44] Andreae M.O. (2019). Emission of trace gases and aerosols from biomass burning–an updated assessment. Atmos. Chem. Phys..

[45] Jankovic J., Jones W., Burkhart J., Noonan G. (1991). Environmental study of firefighters. Ann. Occup. Hyg..

[46] Kinnes G.M., Hine G.A. (1998). Respiratory Hazards Associated with Fire Investigations (Bureau of Alcohol, Tobacco, and Firearms).

[47] Lowry W.T., Juarez L., Petty C.S., Roberts B. (1985). Studies of toxic gas production during actual structural fires in the Dallas area. J. Forensic. Sci..

[48] Burgess J.L., Nanson C.J., Bolstad-Johnson D.M., Gerkin R., Hysong T.A., Lantz R.C., Sherrill D.L., Crutchfield C.D., Quan S.F., Bernard A.M. (2001). Adverse respiratory effects following overhaul in firefighters. J. Occup. Environ. Med..

[49] Allonneau A., Mercier S., Rieunier F., Menguy-Fleuriot A., Louyot C., Duvollet M., Burlaton G., Nicolas A., Jouffroy R., Prunet B. (2022). Exposure to fire smoke in fire training structures: A prospective observational study. Arch. Environ. Occup. Health.

[50] IARC (1999). IARC Monographs on the Evaluation of Carcinogenic Risks to Humans Volume 71: Re-Evaluation of Some Organic Chemicals, Hydrazine and Hydrogen Peroxide.

[51] Quast J.F. (2002). Two-year toxicity and oncogenicity study with acrylonitrile incorporated in the drinking water of rats. Toxicol. Lett..

[52] Maltoni C., Ciliberti A., Cotti G., Perino G. (1988). Long-term carcinogenicity bioassays on acrylonitrile administered by inhalation and by ingestion to Sprague-Dawley rats. Ann. N. Y. Acad. Sci..

[53] NTP (2021). Acrylonitrile.

[54] Fent K.W., Evans D.E. (2011). Assessing the risk to firefighters from chemical vapors and gases during vehicle fire suppression. J Environ Monit.

[55] Garcia E., Hurley S., Nelson D.O., Hertz A., Reynolds P. (2015). Hazardous air pollutants and breast cancer risk in California teachers: A cohort study. Environ. Health.

[56] Glass D.C., Heyworth J., Thomson A.K., Peters S., Saunders C., Fritschi L. (2015). Occupational exposure to solvents and risk of breast cancer. Am. J. Ind. Med..

[57] IOM (2012). Breast Cancer and the Environment: A Life Course Approach.

[58] Laouali N., Pilorget C., Cyr D., Neri M., Kaerlev L., Sabroe S., Gorini G., Richiardi L., Morales-Suárez-Varela M., Llopis-Gonzalez A. (2018). Occupational exposure to organic solvents and risk of male breast cancer: A European multicenter case-control study. Scand. J. Work Environ. Health.

[59] NTP (2021). Benzene.

[60] IARC (2018). Monographs on the Evaluation of Carcinogenic Risks to Humans, Volume 120: Benzene.

[61] Maltoni C., Conti B., Perino G., Di Maio V. (1988). Further evidence of benzene carcinogenicity. Results on Wistar rats and Swiss mice treated by ingestion. Ann. N. Y. Acad. Sci..

[62] Maltoni C., Conti B., Cotti G., Belpoggi F. (1985). Experimental studies on benzene carcinogenicity at the Bologna Institute of Oncology: Current results and ongoing research. Am. J. Ind. Med..

[63] Fent K.W., Toennis C., Sammons D., Robertson S., Bertke S., Calafat A.M., Pleil J.D., Wallace M.A.G., Kerber S., Smith D.L. (2019). Firefighters’ and instructors’ absorption of PAHs and benzene during training exercises. Int. J. Hyg. Environ. Health.

[64] Fent K.W., Eisenberg J., Snawder J., Sammons D., Pleil J.D., Stiegel M.A., Mueller C., Horn G.P., Dalton J. (2014). Systemic Exposure to PAHs and Benzene in Firefighters Suppressing Controlled Structure Fires. Ann. Occup. Hyg..

[65] Wallace M.A.G., Pleil J.D., Oliver K.D., Whitaker D.A., Mentese S., Fent K.W., Horn G.P. (2019). Targeted GC-MS analysis of firefighters’ exhaled breath: Exploring biomarker response at the individual level. J. Occup. Environ. Hyg..

[66] Fent K.W., Toennis C., Sammons D., Robertson S., Bertke S., Calafat A.M., Pleil J.D., Wallace M.A.G., Kerber S., Smith D. (2020). Firefighters’ absorption of PAHs and VOCs during controlled residential fires by job assignment and fire attack tactic. J. Expo. Sci. Environ. Epidemiol..

[67] Fent K.W., Evans D.E., Booher D., Pleil J.D., Stiegel M.A., Horn G.P., Dalton J. (2015). Volatile Organic Compounds Off-gassing from Firefighters’ Personal Protective Equipment Ensembles after Use. J Occup Environ Hyg.

[68] Rosting C., Olsen R. (2020). Biomonitoring of the benzene metabolite s-phenylmercapturic acid and the toluene metabolite s-benzylmercapturic acid in urine from firefighters. Toxicol. Lett..

[69] Laitinen J., Mäkelä M., Mikkola J., Huttu I. (2010). Fire fighting trainers’ exposure to carcinogenic agents in smoke diving simulators. Toxicol. Lett..

[70] Fent K.W., Mayer A.C., Toennis C., Sammons D., Robertson S., Chen I.C., Bhandari D., Blount B.C., Kerber S., Smith D.L. (2022). Firefighters’ urinary concentrations of VOC metabolites after controlled-residential and training fire responses. Int. J. Hyg. Environ. Health.

[71] Fent K.W., Evans D.E., Babik K., Striley C., Bertke S., Kerber S., Smith D., Horn G.P. (2018). Airborne contaminants during controlled residential fires. J. Occup. Environ. Hyg..

[72] Brandt-Rauf P.W., Fallon L.F., Tarantini T., Idema C., Andrews L. (1988). Health hazards of fire fighters: Exposure assessment. Br. J. Ind. Med..

[73] Kirk K.M., Splawinski Z., Bott R.C., Logan M.B. (2021). Combustion products generated in simulated industrial fires. J. Occup. Environ. Hyg..

[74] Kirk K.M., Logan M.B. (2015). Structural Fire Fighting Ensembles: Accumulation and Off-gassing of Combustion Products. J. Occup. Environ. Hyg..

[75] Pleil J.D., Stiegel M.A., Fent K.W. (2014). Exploratory breath analyses for assessing toxic dermal exposures of firefighters during suppression of structural burns. J. Breath Res..

[76] Austin C.C., Wang D., Ecobichon D.J., Dussault G. (2001). Characterization of volatile organic compounds in smoke at municipal structural fires. J. Toxicol. Environ. Health Part A.

[77] Austin C.C., Wang D., Ecobichon D.J., Dussault G. (2001). Characterization of volatile organic compounds in smoke at experimental fires. J. Toxicol. Environ. Health Part A.

[78] Treitman R.D., Burgess W.A., Gold A. (1980). Air contaminants encountered by firefighters. Am. Ind. Hyg. Assoc. J..

[79] Alharbi B.H., Pasha M.J., Al-Shamsi M.A.S. (2021). Firefighter exposures to organic and inorganic gas emissions in emergency residential and industrial fires. Sci. Total Environ..

[80] Kirk K.M., Logan M.B. (2019). Exposures to air contaminants in compartment fire behavior training (CFBT) using particleboard fuel. J. Occup. Environ. Hyg..

[81] Hill T.A., Siedle A.R., Perry R. (1972). Chemical hazards of a fire-fighting training environment. Am. Ind. Hyg. Assoc. J..

[82] Reinhardt T., Ottmar R. (2000). Smoke Exposure at Western Wildfires.

[83] Reinhardt T., Ottmar R., Hanneman A. (2000). Smoke Exposure among Firefighters at Prescribed Burns in the Pacific Northwest.

[84] Navarro K.M., West M.R., O’dell K., Sen P., Chen I.-C., Fischer E.V., Hornbrook R.S., Apel E.C., Hills A.J., Jarnot A. (2021). Exposure to Particulate Matter and Estimation of Volatile Organic Compounds across Wildland Firefighter Job Tasks. Environ. Sci. Technol..

[85] Sathiakumar N., Delzell E. (2009). A follow-up study of mortality among women in the North American synthetic rubber industry. J. Occup. Environ. Med..

[86] IARC (2008). Monographs on the Evaluation of Carcinogenic Risks to Humans, Volume 97: 1,3-Butadiene, Ethylene Oxide and Vinyl Halides (Vinyl Fluoride, Vinyl Chloride and Vinyl Bromide).

[87] NTP (2021). 1,3-Butadiene.

[88] Sjöström M., Julander A., Strandberg B., Lewné M., Bigert C. (2019). Airborne and Dermal Exposure to Polycyclic Aromatic Hydrocarbons, Volatile Organic Compounds, and Particles among Firefighters and Police Investigators. Ann. Work Expo. Health.

[89] Warner M., Mocarelli P., Samuels S., Needham L., Brambilla P., Eskenazi B. (2011). Dioxin exposure and cancer risk in the Seveso Women’s Health Study. Environ. Health Perspect..

[90] Koual M., Cano-Sancho G., Bats A.-S., Tomkiewicz C., Kaddouch-Amar Y., Douay-Hauser N., Ngo C., Bonsang H., Deloménie M., Lecuru F. (2019). Associations between persistent organic pollutants and risk of breast cancer metastasis. Environ. Int..

[91] VoPham T., Bertrand K.A., Jones R.R., Deziel N.C., DuPré N.C., James P., Liu Y., Vieira V.M., Tamimi R.M., Hart J.E. (2020). Dioxin exposure and breast cancer risk in a prospective cohort study. Environ. Res..

[92] Danjou A.M.N., Coudon T., Praud D., Lévêque E., Faure E., Salizzoni P., Le Romancer M., Severi G., Mancini F.R., Leffondré K. (2019). Long-term airborne dioxin exposure and breast cancer risk in a case-control study nested within the French E3N prospective cohort. Environ. Int..

[93] IARC (2012). Monographs on the Evaluation of Carcinogenic Risks to Humans, Volume 100F: 2,3,7,8-Tetrachlorodibenzopara-Dioxin, 2,3,4,7,8-Pentachlorodibenzofuran, and 3,3′,4,4′,5-Pentachlorobiphenyl.

[94] NTP (2006). TR 525: Toxicology and Carcinogenesis Studies of 1,3,4,7,8-Pentachlorodibenzofuran in Female Sprague-Dawley Rats.

[95] Mayer A.C., Fent K.W., Chen I.-C., Sammons D., Toennis C., Robertson S., Kerber S., Horn G.P., Smith D.L., Calafat A.M. (2021). Characterizing exposures to flame retardants, dioxins, and furans among firefighters responding to controlled residential fires. Int. J. Hyg. Environ. Health.

[96] Hsu J.F., Guo H.R., Wang H.W., Liao C.K., Liao P.C. (2011). An occupational exposure assessment of polychlorinated dibenzo-p-dioxin and dibenzofurans in firefighters. Chemosphere.

[97] Shaw S.D., Berger M.L., Harris J.H., Yun S.H., Wu Q., Liao C., Blum A., Stefani A., Kannan K. (2013). Persistent organic pollutants including polychlorinated and polybrominated dibenzo-p-dioxins and dibenzofurans in firefighters from Northern California. Chemosphere.

[98] Chernyak Y.I., Shelepchikov A.A., Brodsky E.S., Grassman J.A. (2012). PCDD, PCDF, and PCB exposure in current and former firefighters from Eastern Siberia. Toxicol. Lett..

[99] Organtini K.L., Myers A.L., Jobst K.J., Reiner E.J., Ross B., Ladak A., Mullin L., Stevens D., Dorman F.L. (2015). Quantitative Analysis of Mixed Halogen Dioxins and Furans in Fire Debris Utilizing Atmospheric Pressure Ionization Gas Chromatography-Triple Quadrupole Mass Spectrometry. Anal. Chem..

[100] Ruokojärvi P., Aatamila M., Ruuskanen J. (2000). Toxic chlorinated and polyaromatic hydrocarbons in simulated house fires. Chemosphere.

[101] Holmes A.K., Koller K.R., Kieszak S.M., Sjodin A., Calafat A.M., Sacco F.D., Varner D.W., Lanier A.P., Rubin C.H. (2014). Case-control study of breast cancer and exposure to synthetic environmental chemicals among Alaska Native women. Int. J. Circumpolar Health.

[102] He Y., Peng L., Zhang W., Liu C., Yang Q., Zheng S., Bao M., Huang Y., Wu K. (2018). Adipose tissue levels of polybrominated diphenyl ethers and breast cancer risk in Chinese women: A case-control study. Environ. Res..

[103] Hurley S., Reynolds P., Goldberg D., Nelson D.O., Jeffrey S.S., Petreas M. (2011). Adipose levels of polybrominated diphenyl ethers and risk of breast cancer. Breast Cancer Res. Treat..

[104] Tonxnet Chemical Carcinogenesis Research Information System: United States National Library of Medicine; 2016. https://www.ncbi.nlm.nih.gov/pcsubstance?term=%22Chemical%20Carcinogenesis%20Research%20Information%20System%20(CCRIS)%22%5BSourceName%5D%20AND%20hasnohold%5Bfilt%5D.

[105] Li Z.-H., Liu X.-Y., Wang N., Chen J.-S., Chen Y.-H., Huang J.-T., Su C.-H., Xie F., Yu B., Chen D.-J. (2012). Effects of decabrominated diphenyl ether (PBDE-209) in regulation of growth and apoptosis of breast, ovarian, and cervical cancer cells. Environ. Health Perspect..

[106] Mercado-Feliciano M., Bigsby R.M. (2008). The polybrominated diphenyl ether mixture DE-71 is mildly estrogenic. Environ. Health Perspect..

[107] Kodavanti P.R.S., Coburn C.G., Moser V.C., MacPhail R.C., Fenton S.E., Stoker T.E., Rayner J.L., Kannan K., Birnbaum L.S. (2010). Developmental exposure to a commercial PBDE mixture, DE-71: Neurobehavioral, hormonal, and reproductive effects. Toxicol. Sci..

[108] Park J.-S., Voss R.W., McNeel S., Wu N., Guo T., Wang Y., Israel L., Das R., Petreas M. (2015). High Exposure of California Firefighters to Polybrominated Diphenyl Ethers. Environ. Sci. Technol..

[109] Ekpe O.D., Sim W., Choi S., Choo G., Oh J.-E. (2021). Assessment of Exposure of Korean Firefighters to Polybrominated Diphenyl Ethers and Polycyclic Aromatic Hydrocarbons via Their Measurement in Serum and Polycyclic Aromatic Hydrocarbon Metabolites in Urine. Environ. Sci. Technol..

[110] Shen B., Whitehead T.P., McNeel S., Brown F.R., Dhaliwal J., Das R., Israel L., Park J.-S., Petreas M. (2015). High levels of polybrominated diphenyl ethers in vacuum cleaner dust from California fire stations. Environ. Sci. Technol..

[111] Banks A.P.W., Engelsman M., He C., Wang X., Mueller J.F. (2020). The occurrence of PAHs and flame-retardants in air and dust from Australian fire stations. J. Occup. Environ. Hyg..

[112] Levasseur J.L., Hoffman K., Herkert N.J., Cooper E., Hay D., Stapleton H.M. (2022). Characterizing firefighter’s exposure to over 130 SVOCs using silicone wristbands: A pilot study comparing on-duty and off-duty exposures. Sci. Total Environ..

[113] Easter E., Lander D., Huston T. (2016). Risk assessment of soils identified on firefighter turnout gear. J. Occup. Environ. Hyg..

[114] Alexander B.M., Baxter C.S. (2016). Flame-retardant contamination of firefighter personal protective clothing—A potential health risk for firefighters. J. Occup. Environ. Hyg..

[115] Banks A.P.W., Wang X., Engelsman M., He C., Osorio A.F., Mueller J.F. (2021). Assessing decontamination and laundering processes for the removal of polycyclic aromatic hydrocarbons and flame retardants from firefighting uniforms. Environ. Res..

[116] Banks A.P.W., Wang X., He C., Gallen M., Thomas K.V., Mueller J.F. (2021). Off-Gassing of Semi-Volatile Organic Compounds from Fire-Fighters’ Uniforms in Private Vehicles-A Pilot Study. Int. J. Environ. Res. Public Health.

[117] Mayer A.C., Fent K.W., Bertke S., Horn G.P., Smith D.L., Kerber S., La Guardia M.J. (2019). Firefighter hood contamination: Efficiency of laundering to remove PAHs and FRs. J. Occup. Environ. Hyg..

[118] NTP (1996). TR-452: Toxicology and Carcinogenesis Studies of 2,2-Bis(bromomethyl)-l,3-Propanediol in F344/N Rats and B6C3F1 Mice (Feed Studies).

[119] Brown F.R., Whitehead T.P., Park J.-S., Metayer C., Petreas M.X. (2014). Levels of non-polybrominated diphenyl ether brominated flame retardants in residential house dust samples and fire station dust samples in California. Environ. Res..

[120] Trowbridge J., Gerona R., McMaster M., Ona K., Clarity C., Bessonneau V., Rudel R., Buren H., Morello-Frosch R. (2021). Organophosphate and Organohalogen Flame-Retardant Exposure and Thyroid Hormone Disruption in a Cross-Sectional Study of Female Firefighters and Office Workers from San Francisco. Environ. Sci. Technol..

[121] Jayatilaka N.K., Restrepo P., Davis Z., Vidal M., Calafat A.M., Ospina M. (2019). Quantification of 16 urinary biomarkers of exposure to flame retardants, plasticizers, and organophosphate insecticides for biomonitoring studies. Chemosphere.

[122] NTP (2021). Isoprene.

[123] NTP (1999). TR-486: Toxicology and Carcinogenesis Studies of Isoprene in F344/N Rats (Inhalation Studies).

[124] Fent K.W., LaGuardia M., Luellen D., McCormick S., Mayer A., Chen I.C., Kerber S., Smith D., Horn G.P. (2020). Flame retardants, dioxins, and furans in air and on firefighters’ protective ensembles during controlled residential firefighting. Environ. Int..

[125] Bonefeld-Jorgensen E.C., Long M., Fredslund S.O., Bossi R., Olsen J. (2014). Breast cancer risk after exposure to perfluorinated compounds in Danish women: A case-control study nested in the Danish National Birth Cohort. Cancer Causes Control..

[126] Mancini F.R., Cano-Sancho G., Gambaretti J., Marchand P., Boutron-Ruault M., Severi G., Arveux P., Antignac J., Kvaskoff M. (2020). Perfluorinated alkylated substances serum concentration and breast cancer risk: Evidence from a nested case-control study in the French E3N cohort. Int. J. Cancer..

[127] Bonefeld-Jorgensen E.C., Long M., Bossi R., Ayotte P., Asmund G., Krüger T., Ghisari M., Mulvad G., Kern P., Nzulumiki P. (2011). Perfluorinated compounds are related to breast cancer risk in Greenlandic Inuit: A case control study. Environ. Health.

[128] National Academies of Sciences Engineering and Medicine (2022). Guidance on PFAS Exposure, Testing, and Clinical Follow-Up.

[129] White S.S., Calafat A.M., Kuklenyik Z., Villanueva L., Zehr R.D., Helfant L., Strynar M.J., Lindstrom A.B., Thibodeaux J.R., Wood C. (2007). Gestational PFOA exposure of mice is associated with altered mammary gland development in dams and female offspring. Toxicol. Sci..

[130] Yang C., Tan Y.S., Harkema J.R., Haslam S.Z. (2009). Differential effects of peripubertal exposure to perfluorooctanoic acid on mammary gland development in C57Bl/6 and Balb/c mouse strains. Reprod. Toxicol..

[131] New Jersey DWQI Health Effects Subcommittee (2016). Public Review Draft of Health-Based Maximum Contaminant Level Support Document: Perfluoroooctanoic Acid (PFOA).

[132] U.S. EPA (2016). Drinking Water Health Advisory for Perfluorooctanoic Acid (PFOA).

[133] Sibinski L. (1987). Two Year Oral (Diet) Toxicity/Carcinogenicity Study of Fluorochemical FC-143 in Rats.

[134] European Food Safety Authority (2008). Opinion of the Scientific Panel on Contaminants in the Food chain on Perfluorooctane sulfonate (PFOS), perfluorooctanoic acid (PFOA) and their salts. EFSA J..

[135] Laitinen J.A., Koponen J., Koikkalainen J., Kiviranta H. (2014). Firefighters’ exposure to perfluoroalkyl acids and 2-butoxyethanol present in firefighting foams. Toxicol. Lett..

[136] Trowbridge J., Gerona R.R., Lin T., Rudel R.A., Bessonneau V., Buren H., Morello-Frosch R. (2020). Exposure to Perfluoroalkyl Substances in a Cohort of Women Firefighters and Office Workers in San Francisco. Environ. Sci. Technol..

[137] Graber J.M., Black T.M., Shah N.N., Caban-Martinez A.J., Lu S.-E., Brancard T., Yu C.H., Turyk M.E., Black K., Steinberg M.B. (2021). Prevalence and Predictors of Per- and Polyfluoroalkyl Substances (PFAS) Serum Levels among Members of a Suburban US Volunteer Fire Department. Int. J. Environ. Res. Public Health.

[138] Dobraca D., Israel L., McNeel S., Voss R., Wang M., Gajek R., Park J.S., Harwani S., Barley F., She J. (2015). Biomonitoring in California firefighters: Metals and perfluorinated chemicals. J. Occup. Environ. Med..

[139] Barton K.E., Starling A.P., Higgins C.P., McDonough C.A., Calafat A.M., Adgate J.L. (2020). Sociodemographic and behavioral determinants of serum concentrations of per- and polyfluoroalkyl substances in a community highly exposed to aqueous film-forming foam contaminants in drinking water. Int. J. Hyg. Environ. Health.

[140] Jin C., Sun Y., Islam A., Qian Y., Ducatman A. (2011). Perfluoroalkyl acids including perfluorooctane sulfonate and perfluorohexane sulfonate in firefighters. J. Occup. Environ. Med..

[141] Rotander A., Toms L.M., Aylward L., Kay M., Mueller J.F. (2015). Elevated levels of PFOS and PFHxS in firefighters exposed to aqueous film forming foam (AFFF). Environ. Int..

[142] Young A.S., Sparer-Fine E.H., Pickard H.M., Sunderland E.M., Peaslee G.F., Allen J.G. (2021). Per- and polyfluoroalkyl substances (PFAS) and total fluorine in fire station dust. J. Expo. Sci. Environ. Epidemiol..

[143] Muensterman D.J., Titaley I.A., Peaslee G.F., Minc L.D., Cahuas L., Rodowa A.E., Horiuchi Y., Yamane S., Fouquet T.N., Kissel J.C. (2022). Disposition of Fluorine on New Firefighter Turnout Gear. Environ. Sci. Technol..

[144] Labrèche F., Goldberg M.S., Valois M.F., Nadon L. (2010). Postmenopausal breast cancer and occupational exposures. Occup. Environ. Med..

[145] Crew K.D., Gammon M.D., Terry M.B., Zhang F.F., Zablotska L.B., Agrawal M., Shen J., Long C.-M., Eng S.M., Sagiv S.K. (2007). Polymorphisms in nucleotide excision repair genes, polycyclic aromatic hydrocarbon-DNA adducts, and breast cancer risk. Cancer Epidemiol. Biomark. Prev..

[146] McCarty K.M., Santella R.M., Steck S.E., Cleveland R.J., Ahn J., Ambrosone C.B., North K., Sagiv S.K., Eng S.M., Teitelbaum S.L. (2009). PAH-DNA adducts, cigarette smoking, GST polymorphisms, and breast cancer risk. Environ. Health Perspect..

[147] Mordukhovich I., Rossner P., Terry M.B., Santella R., Zhang Y.J., Hibshoosh H., Memeo L., Mansukhani M., Long C.-M., Garbowski G. (2010). Associations between polycyclic aromatic hydrocarbon-related exposures and p53 mutations in breast tumors. Environ. Health Perspect..

[148] Shen J., Terry M.B., Gammon M.D., Gaudet M.M., Teitelbaum S.L., Eng S.M., Sagiv S.K., Neugut A.I., Santella R.M. (2006). IGHMBP2 Thr671Ala polymorphism might be a modifier for the effects of cigarette smoking and PAH-DNA adducts to breast cancer risk. Breast Cancer Res. Treat..

[149] White A.J., Chen J., McCullough L.E., Xu X., Cho Y.H., Teitelbaum S.L., Neugut A.I., Terry M.B., Hibshoosh H., Santella R.M. (2015). Polycyclic aromatic hydrocarbon (PAH)-DNA adducts and breast cancer: Modification by gene promoter methylation in a population-based study. Cancer Causes Control.

[150] Shen J., Liao Y., Hopper J.L., Goldberg M., Santella R.M., Terry M.B. (2017). Dependence of cancer risk from environmental exposures on underlying genetic susceptibility: An illustration with polycyclic aromatic hydrocarbons and breast cancer. Br. J. Cancer.

[151] Hoppe-Jones C., Griffin S.C., Gulotta J.J., Wallentine D.D., Moore P.K., Beitel S.C., Flahr L.M., Zhai J., Zhou J.J., Littau S.R. (2021). Evaluation of fireground exposures using urinary PAH metabolites. J. Expo. Sci. Environ. Epidemiol..

[152] Adetona O., Simpson C.D., Li Z., Sjodin A., Calafat A.M., Naeher L.P. (2017). Hydroxylated polycyclic aromatic hydrocarbons as biomarkers of exposure to wood smoke in wildland firefighters. J. Expo. Sci. Environ. Epidemiol..

[153] Oliveira M., Slezakova K., Alves M.J., Fernandes A., Teixeira J.P., Delerue-Matos C., Pereira M.D.C., Morais S. (2016). Firefighters’ exposure biomonitoring: Impact of firefighting activities on levels of urinary monohydroxyl metabolites. Int. J. Hyg. Environ. Health.

[154] Keir J.L.A., Akhtar U.S., Matschke D.M.J., Kirkham T.L., Chan H.M., Ayotte P., White P.A., Blais J.M. (2017). Elevated Exposures to Polycyclic Aromatic Hydrocarbons and Other Organic Mutagens in Ottawa Firefighters Participating in Emergency, On-Shift Fire Suppression. Environ. Sci. Technol..

[155] Fernando S., Shaw L., Shaw D., Gallea M., VandenEnden L., House R., Verma D.K., Britz-McKibbin P., McCarry B.E. (2016). Evaluation of Firefighter Exposure to Wood Smoke during Training Exercises at Burn Houses. Environ. Sci. Technol..

[156] Rossbach B., Wollschläger D., Letzel S., Gottschalk W., Muttray A. (2020). Internal exposure of firefighting instructors to polycyclic aromatic hydrocarbons (PAH) during live fire training. Toxicol. Lett..

[157] Banks A.P.W., Thai P., Engelsman M., Wang X., Osorio A.F., Mueller J.F. (2021). Characterising the exposure of Australian firefighters to polycyclic aromatic hydrocarbons generated in simulated compartment fires. Int. J. Hyg. Environ. Health.

[158] Keir J.L.A., Akhtar U.S., Matschke D.M.J., White P.A., Kirkham T.L., Chan H.M., Blais J.M. (2020). Polycyclic aromatic hydrocarbon (PAH) and metal contamination of air and surfaces exposed to combustion emissions during emergency fire suppression: Implications for firefighters’ exposures. Sci Total Environ.

[159] Wingfors H., Nyholm J.R., Magnusson R., Wijkmark C.H. (2018). Impact of Fire Suit Ensembles on Firefighter PAH Exposures as Assessed by Skin Deposition and Urinary Biomarkers. Ann. Work Expo. Health.

[160] Stec A.A., Dickens K.E., Salden M., Hewitt F.E., Watts D.P., Houldsworth P.E., Martin F.L. (2018). Occupational Exposure to Polycyclic Aromatic Hydrocarbons and Elevated Cancer Incidence in Firefighters. Sci. Rep..

[161] Oliveira M., Costa S., Vaz J., Fernandes A., Slezakova K., Delerue-Matos C., Teixeira J.P., Pereira M.C., Morais S. (2020). Firefighters exposure to fire emissions: Impact on levels of biomarkers of exposure to polycyclic aromatic hydrocarbons and genotoxic/oxidative-effects. J. Hazard Mater..

[162] Baum J.L., Bakali U., Killawala C., Santiago K.M., Dikici E., Kobetz E.N., Solle N.S., Deo S., Bachas L., Daunert S. (2020). Evaluation of silicone-based wristbands as passive sampling systems using PAHs as an exposure proxy for carcinogen monitoring in firefighters: Evidence from the firefighter cancer initiative. Ecotoxicol. Environ. Saf..

[163] Caban-Martinez A.J.D., Louzado-Feliciano P., Santiago K.M., Baum J.B., Solle N.S., Rivera G., Miric M.M., Perez-Then E.M., Kobetz-Kerman E.N., Daunert S. (2020). Objective Measurement of Carcinogens Among Dominican Republic Firefighters Using Silicone-Based Wristbands. J. Occup. Environ. Med..

[164] Poutasse C.M., Haddock C.K., Poston W.S., Jahnke S.A., Tidwell L.G., Bonner E.M., Hoffman P.D., Anderson K.A. (2022). Firefighter exposures to potential endocrine disrupting chemicals measured by military-style silicone dog tags. Environ. Int..

[165] Strandberg B., Julander A., Sjöström M., Lewné M., Koca Akdeva H., Bigert C. (2018). Evaluation of polyurethane foam passive air sampler (PUF) as a tool for occupational PAH measurements. Chemosphere.

[166] Atlas E.L., Donnelly K.C., Giam C.S., McFarland A.R. (1985). Chemical and biological characterization of emissions from a fireperson training facility. Am. Ind. Hyg. Assoc. J..

[167] Bakali U., Baum J.L., Killawala C., Kobetz E.N., Solle N.S., Deo S.K., Caban-Martinez A.J., Bachas L.G., Daunert S. (2021). Mapping carcinogen exposure across urban fire incident response arenas using passive silicone-based samplers. Ecotoxicol. Environ. Saf..

[168] Abrard S., Bertrand M., De Valence T., Schaupp T. (2019). French firefighters exposure to Benzo[a]pyrene after simulated structure fires. Int. J. Hyg. Environ. Health.

[169] Feunekes F.D., Jongeneelen F.J., vd Laan H., Schoonhof F.H. (1997). Uptake of polycyclic aromatic hydrocarbons among trainers in a fire-fighting training facility. Am. Ind. Hyg. Assoc. J..

[170] Sparer E.H., Prendergast D.P., Apell J.N., Bartzak M.R., Wagner G.R., Adamkiewicz G., Hart J.E., Sorensen G. (2017). Assessment of Ambient Exposures Firefighters Encounter While at the Fire Station: An Exploratory Study. J. Occup. Environ. Med..

[171] Bott R.C., Kirk K.M., Logan M.B., Reid D.A. (2017). Diesel particulate matter and polycyclic aromatic hydrocarbons in fire stations. Environ. Sci. Process Impacts.

[172] Alexander B.M., Baxter C.S. (2014). Plasticizer Contamination of Firefighter Personal Protective Clothing—A Potential Factor in Increased Health Risks in Firefighters. J. Occup. Environ. Hyg..

[173] Navarro K.M., Cisneros R., Schweizer D., Chowdhary P., Noth E.M., Balmes J.R., Hammond S.K. (2019). Incident command post exposure to polycyclic aromatic hydrocarbons and particulate matter during a wildfire. J. Occup. Environ. Hyg..

[174] Baxter C.S., Hoffman J.D., Knipp M.J., Reponen T., Haynes E.N. (2014). Exposure of firefighters to particulates and polycyclic aromatic hydrocarbons. J. Occup. Environ. Hyg..

[175] Robinson M.S., Anthony T.R., Littau S.R., Herckes P., Nelson X., Poplin G.S., Burgess J.L. (2008). Occupational PAH exposures during prescribed pile burns. Ann. Occup. Hyg..

[176] Navarro K.M., Cisneros R., Noth E.M., Balmes J.R., Hammond S.K. (2017). Occupational Exposure to Polycyclic Aromatic Hydrocarbon of Wildland Firefighters at Prescribed and Wildland Fires. Environ. Sci. Technol..

[177] Lauby-Secretan B., Loomis D., Grosse Y., El Ghissassi F., Bouvard V., Benbrahim-Tallaa L., Guha N., Baan R., Mattock H., Straif K. (2013). Carcinogenicity of polychlorinated biphenyls and polybrominated biphenyls. Lancet Oncol..

[178] IARC (2016). Monographs on the Evaluation of Carcinogenic Risks to Humans, Volume 107: Polychlorinated Biphenyls and Polybrominated Biphenyls.

[179] Huang W., He Y., Xiao J., Huang Y., Li A., He M., Wu K. (2019). Risk of breast cancer and adipose tissue concentrations of polychlorinated biphenyls and organochlorine pesticides: A hospital-based case-control study in Chinese women. Environ. Sci. Pollut. Res. Int..

[180] Mayes B.A., Mc Connell E.E., Neal B.H., Brunner M.J., Hamilton S.B., Peters A.C., Ryan M.J., Toft J.D., Singer A.W., Brown J.F. (1998). Comparative carcinogenicity in Sprague-Dawley rats of the polychlorinated biphenyl mixtures Aroclors **1016**, *1242*, 1254, and 1260. Toxicol. Sci..

[181] Martinez J.M., Stephens L.C., Jones L.A. (2005). Long-Term Effects of Neonatal Exposure to Hydroxylated Polychlorinated Biphenyls in the BALB/cCrgl Mouse. Environ. Health Perspect..

[182] Niehoff N.M., Gammon M.D., Keil A.P., Nichols H.B., Engel L.S., Sandler D.P., White A.J. (2019). Airborne mammary carcinogens and breast cancer risk in the Sister Study. Environ. Int..

[183] NTP (2021). Styrene.

[184] IARC (2019). Monographs on the Evaluation of Carcinogenic Risks to Humans, Volume 121: Styrene, Styrene-7,8-Oxide, and Quinoline.

[185] Brody J.G., Moysich K.B., Humblet O., Attfield K.R., Beehler G.P., Rudel R.A. (2007). Environmental pollutants and breast cancer: Epidemiologic studies. Cancer.

[186] Rudel R.A., Attfield K.R., Schifano J.N., Brody J.G. (2007). Chemicals causing mammary gland tumors in animals signal new directions for epidemiology, chemicals testing, and risk assessment for breast cancer prevention. Cancer.

[187] Fent K.W., Alexander B., Roberts J., Robertson S., Toennis C., Sammons D., Bertke S., Kerber S., Smith D., Horn G. (2017). Contamination of firefighter personal protective equipment and skin and the effectiveness of decontamination procedures. J. Occup. Environ. Hyg..

[188] Mayer A.C., Horn G.P., Fent K.W., Bertke S.J., Kerber S., Kesler R.M., Newman H., Smith D.L. (2020). Impact of select PPE design elements and repeated laundering in firefighter protection from smoke exposure. J. Occup. Environ. Hyg..

[189] NCSL (2023). Per- and Polyfluoroalkyl Substances (PFAS) | State Legislation and Federal Action.

[190] Safer States Bill Tracker for Chemical Prioritization/Disclosure/Phase-Out. http://www.saferstates.org/bill-tracker/?states=Minnesota&status=adopted.

[191] (2019). 116th United States Congress. National Defense Authorization Act for Fiscal Year 2020. Public Law 116–92.

[192] International Association of Fire Fighters Presumptive Health Initiative: International Association of Fire Fighters. https://www.iaff.org/presumptive-health/.

[193] IPSDI (2019). Fire Fighter Exposure Tracking App.

[194] IARC (2016). Monographs on the Evaluation of Carcinogenic Risks to Humans, Volume 109: Outdoor Air Pollution.

[195] Zeinomar N., Oskar S., Kehm R.D., Sahebzeda S., Terry M.B. (2020). Environmental exposures and breast cancer risk in the context of underlying susceptibility: A systematic review of the epidemiological literature. Environ. Res..

[196] Kay J.E., Cardona B., Rudel R.A., Vandenberg L.N., Soto A.M., Christiansen S., Birnbaum L.S., Fenton S.E. (2022). Chemical Effects on Breast Development, Function, and Cancer Risk: Existing Knowledge and New Opportunities. Curr. Environ. Health Rep..

[197] Howdeshell K.L., Hotchkiss A.K., Gray L.E. (2017). Cumulative effects of antiandrogenic chemical mixtures and their relevance to human health risk assessment. Int. J. Hyg. Environ. Health.

[198] Kortenkamp A. (2008). Low dose mixture effects of endocrine disrupters: Implications for risk assessment and epidemiology. Int. J. Androl..

